# Detection of Parasites and Parasitic Infections of Free-Ranging Wildlife on a Game Ranch in Zambia: A Challenge for Disease Control

**DOI:** 10.1155/2012/296475

**Published:** 2012-05-31

**Authors:** Hetron Mweemba Munang'andu, Victor M. Siamudaala, Musso Munyeme, King Shimumbo Nalubamba

**Affiliations:** ^1^Section of Aquatic Medicine and Nutrition, Department of Basic Sciences and Aquatic Medicine, Norwegian School of Veterinary Sciences, Ullevålsveien 72, P.O. Box 8146 Dep, 0033, Oslo, Norway; ^2^Kavango Zambezi Transfrontier Conservation Area Secretariat, Kasane 821, Gaborone, Botswana; ^3^Department of Disease Control, School of Veterinary Medicine, University of Zambia, P.O. Box 32379, Lusaka 10101, Zambia; ^4^Department of Clinical Studies, School of Veterinary Medicine, University of Zambia, P.O. Box 32379, Lusaka 10101, Zambia

## Abstract

*Ex-situ* conservancies are expanding alternatives to livestock production in Zambia albeit the lack of information on circulating infectious parasites from wildlife. Therefore, 12 wildlife species were examined on a game ranch were all species were found to be infected by *Rhipecephalus* spp. Haemoparasite infections were estimated at 7.37% (*n* = 95) with *Babesia* spp. detected in bushbuck (*Tragelaphus scriptus*); *Anaplasma marginale* in impala (*Aepyceros melampus*) and puku (*Kobus vardonii*) for the first time in Zambia. The majority of worm species isolated from bovids were not detected in equids and, *vice versa*. Our findings intimate ecological and behavioural patterns of some animals as deterministic to exposure. Kafue lechwe (*Kobus leche kafuensis*) had the widest range of worm species with more infected organs than other animals suggesting their semi aquatic nature contributory to prolonged worm exposure compared to other animals. On the other hand, Kafue lechwe had the least tick infections attributable more to shorter attachment periods as they spend prolonged periods submerged in water. Our findings indicate the vital role that wildlife plays in the epidemiology of parasitic diseases. To reduce the infection burden, control measures should be focused on reducing transmission to highly susceptible animal species as described herein.

## 1. Introduction

*Ex-situ* conservation is expanding in Zambia with the aim of promoting wildlife utilization alongside livestock production. The industry has turned out to be an alternative to cattle ranching given that the latter has been ravaged by tick-borne diseases that have caused a significant decline on the cattle population in Zambia [1, 2]. The shift from cattle ranching to game ranching reduces economic losses incurred in livestock production due to continuous prophylactic treatment of cattle unlike wildlife species that are resistant to tick-borne diseases [3]. Overall, game ranching promotes preservation of different wildlife species by protecting animals from poaching which is rare on the game ranches but common on state-owned national parks. In addition, the involvement of game ranches in stocking endangered species such as the kafue lechwe (*Kafue leche kafuensis*) and Black lechwe currently on the International Union Conservation of Nature (IUCN) red list of threatened species is a good conservation strategy which aims at serving these species from extinction [4]. Besides, the translocation of animals from different ecosystems into one habitat leads to stocking of animals that would, otherwise, have not shared a habitat under natural conditions. The mixing of animals from different ecosystems into one habitat is likely to be a proponent of introducing diseases sourced from different ecosystems into a new habitat thereby exposing animals to parasitic infections they would otherwise have never been exposed to. Hence, there is need to develop trace-back systems that track diseases obtained from different ecosystems. It has become paramount to investigate parasitic diseases of wildlife with a view of generating baseline data for use in trace-back systems during disease outbreaks and the translocation of animals from one ecosystem to the other. In the present study, we investigated the presence of endo, and ectoparasites of different wildlife species reared on a game ranch in central Zambia in order to obtain baseline data on the nature of parasitic infections obtained from wildlife in this part of the country. We also wanted to find out the prevalence levels of different parasitic infections on different wildlife species as a way of identifying control strategies that could be used to reduce parasitic burden on game ranches by advising game ranchers to use control strategies likely to reduce the prolonged survival of vector species engaged in transmission of different diseases. The challenge of developing effective disease control strategies for the control of parasitic infections in wildlife medicine is herein discussed. Although this study is based on survey data obtained from central Zambia, it brings into perspective challenges faced by veterinarians in the control of parasitic diseases given the expansion of game ranching across Africa.

## 2. Materials and Methods

### 2.1. Study Area

 The study was carried out on a game ranch located approximately 45 km northeast of Lusaka. The ranch covers a total area of 4,500 km^2^ and is located at an altitude of 1,100 meters. The mean annual rainfall was about 950 mm while summer temperatures varied between 20°C–32°C in the months of October to March. Winter temperatures varied between 10°C–26°C in the months of June to August. Relative humidity was below 40% throughout the year. Vegetation on the ranch comprised of miombo and acacias woodlands with open savannah grasslands. The ranch encompasses three periannual large dams that provide adequate water for the survival of various species including the semiaquatic kafue lechwe (*Kobus leche kafuensis*) and sitatunga (*Tragelaphus spekii*). Tick and worm infections were controlled by rotational burning of grass in the dry season and use of antihelminths and acaricides administered using Duncan applicators [5–7]. The ranch was surrounded by a 2.5 m fence with a 10-meter fire guard surrounding the entire game ranch.

### 2.2. Animals

The ranch is endowed with several mammalian species comprised of wild ungulates (Table 1), reptiles, and birds. In October 2005 blood samples and smears were collected from a total of 39 animals from six wildlife species captured for translocation (Table 1). The animals were immobilized using M99 (etorphine hydrochloride, Norvatis, Ltd., Johannesburg, South Africa) at standard doses and were later revived using M5050 (revivon, Norvatis, Ltd., Johannesburg, South Africa). In August to October 2004 and July to August 2005, 56 animals from 10 species were sacrificed (killed using a rifle) for parasite infestation and disease surveys (Table 1). Only sacrificed animals (*n* = 56) were used for helminth surveys while all animals (*n* = 95), that is, both the sacrificed and immobilized, were used for blood parasite and tick infestation surveys.

### 2.3. Sampling of Ticks and Blood Smears

Ticks were collected and stored in bottles and transported to the School of Veterinary Medicine at the University of Zambia in Lusaka for identification. Thin blood smears were made from ear veins on site from all animals captured for translocation and those sacrificed for disease investigations (Table 1). Second sets of blood smears were made from buffy coats from blood collected in EDTA soon after arrival at the laboratory at the School of Veterinary Medicine, University of Zambia in Lusaka. For sacrificed animals, impression smears were also made from the prescapular and parotid lymph nodes on site. All slides were observed under the light microscope (×100) after staining with 20% Giemsa stain.

### 2.4. Sampling of Helminths

Components of the digestive system were separately ligated. From each segment, 180 ml of the contents was placed in a bottle and 20 mL of formalin was added to each bottle. Thereafter, the contents were emptied into sedimentation jars at the School of Veterinary Medicine at the University of Zambia in Lusaka. After sedimentation, worms in the supernatant were picked and stored in 10% formalin bottles. The mesentery was separated from the viscera and was carefully inspected for the presence of *Schistosoma* spp. while the veins were cut and squeezed to release the worms. The worms were picked and stored in 10% formalin. The liver and bile ducts were incised as described by Hansen and Perry [8] to check for flukes. The liver was sliced into small pieces and squeezed to let the flukes drop in water containers followed by draining the water through a 500 *μ*m sieve (Endecotts Ltd., England). All flukes were collected and stored in 10% formalin. Other organs inspected were the trachea, lungs, heart, tongue, and skeletal muscles. Recovered worms were put in petri dishes containing lactophenol overnight. Thereafter, worms were identified using standard keys after mounting on glass slides [9].

## 3. Results

### 3.1. Blood Parasites

**Figure 1:**
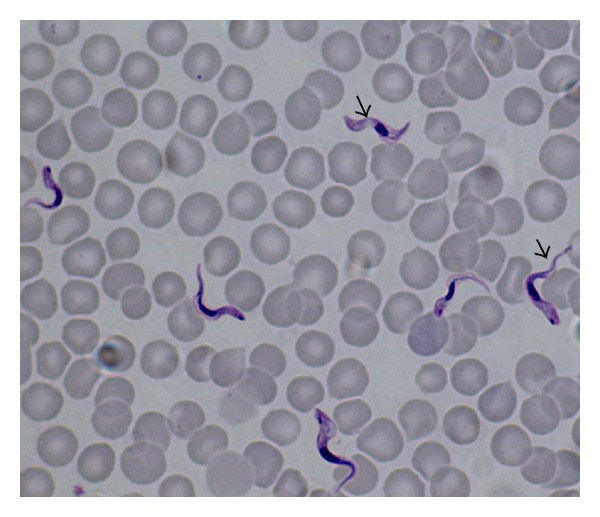
Show detection of *Trypanosoma congolense* (arrow) in greater kudu (*Tragelaphus strepsiceros*).

**Figure 2:**
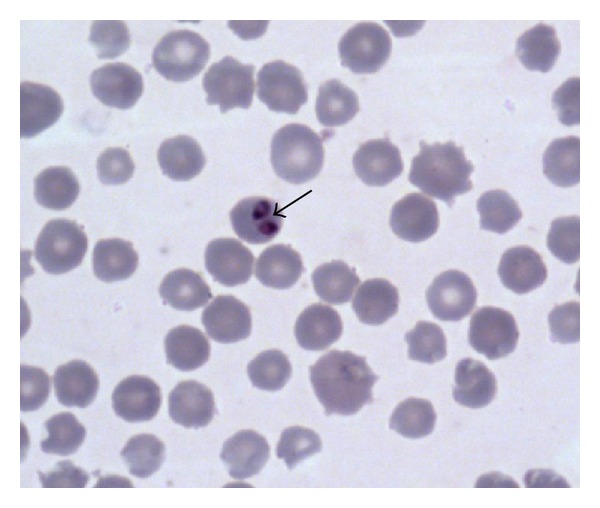
Shows detection *Babesia *spp. in bushbuck (*Tragelaphus scriptus*).

Identification of parasites was based on standard keys [9–11].  Figure 1 shows *Trypanosoma congolense* detected in greater kudu, while Figure 2 shows infection of *Babesia *spp. detected from bushbuck. *Anaplasma marginale* appeared as dense intraerythrocytic rounded bodies located on the edges of red blood cells ranging from 3.21–9.78 *μ*m (*n* = 52) which is in line with observations made elsewhere [9, 12]. As shown in Figure 2,  *Babesia *spp. were characterized by pairs of merozoites in blood smears which is in line with observations made by Homer et al. [10] and Schuster [11] who pointed out that detection of pairs or tetrads of merozites also known as “Maltose cross” in stained red blood cells is characteristic of *Babesia *spp. infection. Overall, our findings show a low prevalence of blood parasite infections on the game ranch. As shown in Table 2, there were only seven animals having blood parasites giving an overall infection rate of 7.37% (*n* = 95). Prevalence rates for individual species of blood parasites were estimates at 2.11% (*n* = 95) for* Anaplasma marginale*, *Babesia *species, and *Trypanosoma congolense* while for Theileria piroplasms the infection rate was estimated at 1.05% (*n* = 95). *Trypanosoma congolence* was only detected in greater kudu at an infection rate of 18.18% (*n* = 11).

### 3.2. Ticks

Infection rates of different tick species for the different animals examined on the game ranch were generally high (Table 3). Some animal species were infected by different tick species while others were only infected by one species. *Rhipicephalus *spp. were the most prevalent tick species infesting all animal species examined (Table 3). *Amblyomma variegatum* was collected from six species while *Hyaloma trancutum* together with other *Hyaloma *spp. were collected from five animal species. Bushbuck, defassa waterbuck (*Kobus ellipsiprymnus*), and wildebeest (*Connochaetes taurinus*) were infested by a wider range of tick species unlike impala, kafue lechwe, reedbuck (*Redunca redunca*), and tsessebe (*Damaliscus lunatus*) which were only infested by *Rhipicephalus appendiculatus*. Kafue lechwe and impala had the least infection rates of 22.7% (*n* = 22) and 33.3% (*n* = 6), respectively. Only two tick control measures were used on the game ranch, namely, the use of Duncan applicators [13] and rotational burning. For rotational burning, the game ranch was divided into four sections and only one section was burnt at a time allowing the animals to graze on the unburnt areas. Duncan applicators were used for the control of ticks by administering acaricide pour-ons on animals. The efficacy of these control measures was not evaluated in the present study.

### 3.3. Helminths

Table 4 shows a list of helminthes detected from 10 animal species examined on the game ranch. Generally, infection rates were high for most animal species (Table 4). Kafue lechwe and Burcelli's zebra (*Equus burchelli*) were infected by a wide range of worm species than other animal species. This can be attributed to the fact that there were more animals examined from these species than other animal species (Table 4). On contrast, defassa waterbuck, tsessebe, and greater kudu were only infected by one helminth species. *Schistosoma *species were only reported from kafue lechwe while *Gastrodiscus aegyptiacus, Gastrophilus meridionatis, Strongylus equines*, and *Strongylus vulgaris* were only reported from zebra (Table 4). *Oesophagosotomum *spp. were the most common worm species infecting both bovids and equids while the amphistomes and paramphistomes were only recorded in bovids. Kafue lechwe had the widest organ distribution of worm infections being infected in seven different organs followed by Burcelli's zebra which had infections in three different organs. It is interesting to note that most worm species were specialized to specific organs despite infecting multiple hosts. For example, *Stillesia hepatica* was only found in the liver of infected kafue lechwe, puku, and greater kudu, while *Gaigeria panchyselis* was only found in the small intestines of puku, kafue lechwe, tsessebe, and impala. The only control measure used was the administering of antihelminthes using Dancun applicators.

## 4. Discussion

Prevalence for haemoparasite infections was generally low despite high-tick infection rates observed on the animals. It is interesting to note that all major tick-borne diseases infecting livestock diseases in Zambia [1, 14] were detected on the game ranch. The low infection rates observed in the present study could be attributed to the detection method used considering that the use of blood smears does not detect previous exposure and that low infection rates can easily be missed using this technique. Hence, it is likely that if we had used more robust diagnostic tools such as molecular-biology-based techniques that are more sensitive, higher infection rates would have been determined. On the other hand, the use of serological assays such as the enzyme linked immunosorbent assay (ELISA) would have determined the seroprevalence for animals previously exposed to haemoparasite infections. We did not find clinical cases at the times of the surveys although we did not analyze the blood samples to determine whether infections by hemoparasites caused changes in blood parameters. Besides, the sample size of the animals examined and the number of animals infected by different blood parasites obtained in the present study were inadequate to carry out analysis on the impact of haemoparasites on blood parameters. However, these findings are consistent with other studies that have shown that detection of blood parasites in wildlife is often incidental. This is supported by observations made by other scientists that wildlife are resistant to haemoparasite infections and that clinical disease is often stress related. Besides, Munang'andu et al. [15, 16] recently reported *Babesia *spp. infections in free ranging pukus and *Trypanosoma brucei* in free ranging greater kudu without clinical disease on game ranches in Zambia. To our knowledge, this is the first report of *Babesia *spp. infections in bushbuck and *Anaplasma marginale* in puku and impala in Zambia. Overall, these findings point to the fact that wildlife could play an important role in the epidemiology of haemoparasites in Zambia. This implies that while tick control using acaricides could be reducing the occurrence of tick-borne diseases in livestock, the expansion of game ranching could have a long-term adverse effect of expanding the reservoir host occupancy range of tick-borne diseases whose spillover into cattle ranching would impact negatively on livestock production. This poses a significant challenge for control of tick-borne diseases especially in interface areas where concurrent expansion of wildlife and livestock production is taking places. However, there is need for detailed epidemiology studies to determine the role of different wildlife species in the epidemiology of these diseases in countries where game ranching is expanding.

Generally, *Rhipicephalus appendiculatus* and *Rhipicephalus *species were the most common tick-species infecting multiple host species. *Amblyomma variegatum* and *Hyalomma trancutum* were collected from fewer animal hosts than *Rhipicephelus appendiculatus*. It is not known whether this difference was based on host preference or the relative abundance of the different tick species on the game ranch. Moreover, some animal species like tsessebe, reedbuck, and impala were only infected by *Rhipicephalus appendiculatus* further showing that *Rhipicephalus appendiculatus* was the most common tick species infecting both the bovids and equids on the game ranch. Kafue lechwe are semiaquatic medium sized antelopes often submerged up to the shoulders sometimes leaving only the nostrils when frightened [17, 18]. Hence, the only foreseeable reason why kafue lechwe had low infection rates is that because of its semiaquatic nature, ticks infecting this animal species are likely to drop-off when the animals are submerged in water thereby reducing the attachment time on the host. However, we observed low infection rates on impala which is purely an on-land species, and not semiaquatic like kafue lechwe, although the sample size obtained in this study was low (*n* = 4, Table 3). We did not establish whether impala is a less favored host for tick infection while species such as the deffasa waterbuck, bushbuck, puku, and greater kudu were not only infected by a wide range of tick species but also had high infection rates for most tick species (Table 2). However, there is need for detailed experimental studies to determine the host preference of tick infections in wildlife and to establish reasons why some animal species are less infested than others. Information obtained from such studies would help in selecting wildlife species for culling especially in situation where population reduction of selected wildlife species is aimed at reducing the tick burden. For example, when tick burden is high, animal species that are more vulnerable to infestation can be reduced by culling or safari hunting while the less infested species are left to increase.

Kafue lechwe and Burchelli's zebra were the most infected by helminthes. In addition, kafue lechwe had the widest organ distribution of infections than other animal species. Elsewhere [8, 19], it has been shown that gastrointestinal worm infection rates are dependent on a number of factors which include the number of infective larvae ingested by the host, which in turn is influenced by climatic factors, vegetation, and animal density. Dry open areas prone to excessive heat are hostile for the survival of infective larvae while moist areas near water sources are conducive for the survival infective larvae. This would account for reasons why Ng'ang'a et al. [19] consistently recovered infective larvae around watering points throughout their study period unlike semiarid open areas that had no infective larvae during the dry season. In their conclusion [19], they noted that watering points were an important source of infection for animals, especially during the dry season when other pastures were noninfective. On moist herbage, larvae of different nematodes migrate up and down the blades of grass which facilitate the uptake of infective larvae by grazing animals. During the dry season, areas around water sources attract more animals for grazing thereby increasing the animal population density. As pointed out by Chingwena et al. [20], animals that aggregate in these places are likely to get infected by infective larvae. This would account for the reason why kafue lechwe had a wide infection rate of different worm species in different organs as a result of constant exposure to infection by grazing on moist pastures that harbor high infection rates of infective larvae around water sources close to their habitats. Moreover, the timing of the current surveys which was in the dry season between August and October when there was scarcity of pasture and water on the game ranch, moist conditions prevailing at water sources indicate that these areas had the highest levels of infective larvae leading to transmission of these larvae to the semiaquatic kafue lechwe that graze around the water sources close to their habitat unlike other animal species found in open dry pastures that are less infective during the dry season. By being definitive host, kafue lechwe are likely to serve as critical determinants of infection to other animals as they contaminate the pastures around the water sources with fecal droppings containing infective larvae. Besides, infective larvae deposited in water by defecating lechwes during times when they are submerged in water are likely to infect other animals that come to drink water. By maintaining an active transmission cycle, kafue lechwe is likely to save as a continuous source of infection to other animal species. These observations indicate that reducing infection to kafue lechwe at water sources is likely to reduce the source of infection to other animals. Hence, these is a need for innovative disease control strategies that would reduce the cycle of transmission between infected pastures at water sources and kafue lechwe in order to reduce worm burden infections of wildlife species reared on game ranches. 

Phiri et al. [21] pointed out that snail intermediate of worm species like *Schistosoma *spp. are often concentrated in marshy areas or marginal shallow water areas of ox-bow lakes, lagoons, and rivers. Animals that aggregate in these places increase the contact between miracidia and snail intermediate hosts. Hamond [22] pointed out that the higher the number of final hosts and snails are found together at one site, the more the likelihood that worm infection will propagate and be transmitted to other species. Hence, kafue lechwe which are final hosts and predominantly occupy marshy areas are likely to maintain a high transmission cycle of *Schistosoma *spp. with snail intermediate hosts found on the edges of water sources on the game ranch. This would account for reasons why kafue lechwe were the only species infected by *Schistoma *spp. on the game ranch.

It is interesting to note that most worm species identified in kafue lechwe in the Kafue basin were also detected in the present study [23]. It is likely that these worms could have been introduced on the game ranch by the first breeding stock that was translocated from the kafue basin. We, therefore, advocate that treatment of animals against parasitic infections and use of pour-on acaricides and antihelminths should be carried out prior to or during translocation to reduce the transmission of parasites from one ecosystem to the other. Some helminthes were isolated from several wildlife hosts while others were limited to single hosts. For example, *Gaigera panchyscelis* was isolated from kafue lechwe, impala, and sable antelopes while *Borrostomum trignocephalum* was only isolated from kafue lechwe. In addition, some worm species were only isolated from the bovids and not the equids. For example, *Stillesia hepatica* was only isolated from greater kudu and puku which are bovids while *Strongylus equinus*, *Strongylus vugaris*, and *Anaplocephala perfoliata* were only isolated from Burchelli's zebra which is an equid. These findings suggest that there is host preference for some worm species. On the contrary, some species were collected from a wide host range suggesting that there is interspecies transmission between different animal hosts. For example, *Oesophagostomum *spp. were found to infect both the bovids and equids.

Although different approaches have been used for control of tick and worm infections in wildlife [5, 6, 24], there has been no comprehensive study that assessed the efficacy of these techniques in Zambia. McGranahan [25] assessed the perceptions of game ranchers on the use of rotational burning as a tick control strategy and observed that there was a low attitude generally as most game ranchers did not understand the effectiveness of this technique. Hence, there is need for a quantitative assessment to determine the efficacy of rotational burning as a tick control strategy in game ranching. The major limiting factor to use of Duncan applicators as a tick control strategy for wildlife is that not all animals on game ranches get in contact with the applicators, and that this technique works better for animals kept in captivity under closed confinements. For free-ranging animals, it is unlikely that all animal will rub contacts with Dancun applicators for animals to get a pouring of the acaricide on their body surfaces. In some cases, the use of livestock as a tick control strategy has been suggested in situations where cattle are allowed to graze on the game ranch and latter dipped in acaricide dip-tanks to get rid of the ticks. Doing this a number of times is expected to reduce the tick-burden as cattles are used to sweep off the tick-population on the game ranch. However, the danger with this technique is the transmission of animal diseases between cattle and wildlife which could spark unexpected disease outbreaks. Observations made from this study clearly show that much as control of parasites and parasitic diseases in livestock and other domestic animal species have reached advanced stages, control measures in wildlife medicine are still in their infancy. Hence, there still remains the challenge of finding the most effective way of controlling tick infection and other parasitic infections of wildlife. Given the rapid expansion of the wildlife industry in Southern African, there is urgent need for more effective innovations that would help reduce disease transmission of various parasitic diseases in wildlife.

## Figures and Tables

**Table 1:** Totals on the game ranch and number examined.

Species	Total on Game Ranch (2005)	Animals examined (*n*)			
		Sacrificed 2004	Sacrificed 2005	Immobilized 2005	Total examined
Bushbuck (*Tragelaphus scriptus*)	57	—	—	4	4
Defassa waterbuck (*Kobus ellipsiprymnus*)	63	3	5	—	8
Greater kudu (*Tragelaphus strepsiceros*)	25	3	3	5	11
Impala (*Aepyceros melampus*)	509	4	2	—	6
Kafue lechwe (*kobus leche kafuensis*)	380	4	4	14	22
Puku (*Kobus vardoni*)	252	4	2	10	16
Reedbuck *(Redunca redunca) *	72	—	—	4	4
Sable antelope (*Hippotragus niger*)	41	2	—	2	4
Tsessebe (*Damaliscus lunatus*)	42	2	—	—	2
Warthog (*Phacochoerus aethiopicus*)	205	4	—	—	4
Wildebeest (*Connochaetes taurinus*)	60	4	2	—	6
Zebra (*Equus burchelli*)	80	5	3	—	8

Totals	1,786	35	21	39	95

**Table 2:** Haemoparasites detected from blood smears.

Wildlife species	Total examined	Number of animals infected with				Totals
		*Theileria piroplasms*	*Babesia *species	*Anaplasma marginale*	*Trypanosoma congolense*	
Bushbuck (*Tragelaphus scriptus*)	4	—	1	—	—	1
Defassa waterbuck (*Kobus ellipsiprymnus*)	8	—	—	—	—	
Greater kudu (*Tragelaphus strepsiceros*)	11	1	—	—	2	3
Impala (*Aepyceros melampus*)	6	—	—	1	—	1
Kafue lechwe (*Kobus leche kafuensis*)	22	—	—	—	—	
Puku (*Kobus vardoni*)	16	—	1	1	—	2
Sable antelope (*Hippotragus niger*)	4	—	—	—	—	
Reedbuck (*Redunca redunca*)	4	—	—	—	—	
Tsessebe (*Damaliscus lunatus*)	2	—	—	—	—	
Warthog (*Phacochoerus aethiopicus*)	4	—	—	—	—	
Wildebeest (*Connochaetes taurinus*)	6	—	—	—	—	
Zebra (*Equus burchelli*)	8	—	—	—	—	

Totals	95	1	2	2	2	7

**Table 3:** Ticks collected from different wildlife species on game ranch.

Wildlife species	(*n*)	Tick species identified (*)
Bushbuck (*Tragelaphus scriptus*)	4	*Rhipiciphelus appendiculatus *(4)*, Rhipiciphelus *species (4)*, Amblyoma variegatum* (3)*, Hyaloma *species (2).
Defassa waterbuck (*Kobus ellipsiprymnus*)	8	*Rhipiciphelus appendiculatus *(8),* Rhipiciphelus *species (6),* Amblyoma variegatum *(7),* Hyaloma trancutum *(3),* Boophilus decoloratus *(4).
Greater kudu (*Tragelaphus strepsiceros*)	11	*Rhipiciphelus appendiculatus *(11), *Rhipiciphelus *species (9),* Amblyoma variegatum *(8),* Amblyoma *species (2),* Hyaloma Trancutum (4), Hyaloma *species (2).
Impala (*Aepyceros melampus*)	6	*Rhipiciphelus appendiculatus *(2).
Kafue lechwe (*Kobus leche kafuensis*)	22	*Rhipiciphelus appendiculatus *(5),
Puku (*Kobus vardoni*)	16	*Rhipiciphelus appendiculatus *(8),* Rhipiciphelus *species (6),* Amblyoma variegatum *(7),* Hyaloma *species (4).
Sable antelope (*Hippotragus niger*)	4	*Rhipiciphelus appendiculatus *(4),* Rhipiciphelus *species (2).
Reedbuck *(Redunca redunca) *	4	*Rhipiciphelus appendiculatus *(4).
Tsessebe (*Damaliscus lunatus*)	2	*Rhipiciphelus appendiculatus *(2).
Warthog (*Phacochoerus aethiopicus*)	4	*Amblyoma variegatum *(4),* Rhicicephalus *spp. (4).
Wildebeest (*Connochaetes taurinus*)	6	*Amblyoma variegatum *(5),* Rhipiciphelus appendiculatus *(6), *Rhicicephalus *spp. (4),* Hyalomma *species (4),
Zebra (*Equus burchelli*)	8	*Rhipiciphelus appendiculatus *(6),* Rhicicephalus *spp. (4)

(*n*) = total of animals examined, (*) = number of infested animals.

**Table 4:** Helminthes isolated from different wildlife species.

Table Species	Animals	Organ examined	Helminth Species	No infected	Percentage
Defassa waterbuck (*Kobus ellipsiprymnus*)	8	Large intestines	*Oesophagosotomum *spp.	6	75.0%

Geater kudu (*Tragelaphus strepsiceros*)	6	liver	*Stillesia hepatica*	4	66.7%

Impala (*Aepyceros melampus*)	6	Small intestines	*Gaigeria panchyselis, *	5	83.3%
		Large intestines	*Oesophagostomun *species,	2	33.3%

Kafue lechwe (*Kobus leche kafuensis*)	8	Liver	*Fasciola gigantica*	8	100.0%
		Mesentery	*Schistosoma *spp.	5	62.5%
		Peritoneum	*Setaria *species	7	87.5%
		Rumen	*Amphistoma *spp*., *	7	87.5%
			*Parampistoma *spp.	7	87.5%
		Abomasum	*Amphistoma *spp.,	7	87.5%
		Small intestines	*Gaigeria panchyselis, *	4	50.0%
			*Borrostomum trignocephalum*	3	37.5
		Large intestines	*Trichuris *spp.,	5	62.5%
			*Oesophagostomum *species	7	87.5%

Puku (*Kobus vardoni*)	6	liver	*Stillesia hepatica*	4	66.7%
		Large intestines	*Oesophagostomum *species	4	66.7%

Sable antelope (*Hippotragus niger*)	2	Small intestines	*Gaigera pachyscelis*	2	100.0%

Tsessebe (*Damaliscus lunatus*)	2	Small intestines	*Gaigera pachyscelis*	2	100.0%

Warthogs (*Phacochoerus aethiopicus*)	4	Lage intestines	*Oesophagosotomum *spp.,	2	50.0%
			*Trichuris *species,	3	75.0%
			*Trichostrongylus *species	3	75.0%

Wildebeest (*Connochaetes taurinus*)	6	Rumen	*Paramphystomes*	4	66.7%

Zebra (*Equus burchelli*)	8	Ceacum,	*Gastrodiscus aegyptiacus, *	5	62.5%
			*Stelizia *species	3	37.5%
			* Gastrophilus meridionatis*	4	50.0%
		Large intestines	*Oesophagostomum *spp.	7	87.5%
			*Strongylus equinus*	4	50.0%
			*Strongylus vulgaris*	4	50.0%
		Small intestines	*Anaplocephala perfoliata*	6	75.0%

Totals	56				
